# P-437. Prevalence of Hepatitis B Co-infection in People with HIV by Birth Cohort– Potential Impact of HBV Vaccination Availability

**DOI:** 10.1093/ofid/ofae631.637

**Published:** 2025-01-29

**Authors:** Claire (So Jeong) Lee, Tarfa I Verinumbe, Anthony Fojo, Joyce L Jones, Jeanne Keruly, LaQuita N Snow, Richard Moore, Mark S Sulkowski, Oluwaseun Falade-Nwulia

**Affiliations:** University of Toronto, Department of Medicine, Toronto, Ontario, Canada; Johns Hopkins University, Baltimore, Maryland; Johns Hopkins University School of Medicine, Baltimore, Maryland; Johns Hopkins University School of Maryland, Baltimore, Maryland; The Johns Hopkins University School of Medicine, Baltimore, MD; Johns Hopkins University, Baltimore, Maryland; Johns Hopkins University, Baltimore, Maryland; Johns Hopkins University School of Medicine, Baltimore, Maryland; Johns Hopkins University, Baltimore, Maryland

## Abstract

**Background:**

Due to shared modes of transmission, people with HIV (PWH) have disproportionately high rates of hepatitis B virus (HBV) infection. Availability of the HBV vaccine in the US since 1982 and recommendations for universal and catch-up vaccination of infants and children since the 1990s may be associated with lower HBV prevalence among PWH born after 1980. To study how HBV vaccination availability impacted co-infection rates in PWH, we compared the prevalence of active HBV infection at entry into an HIV cohort by birth year groups.

**Table 1.**
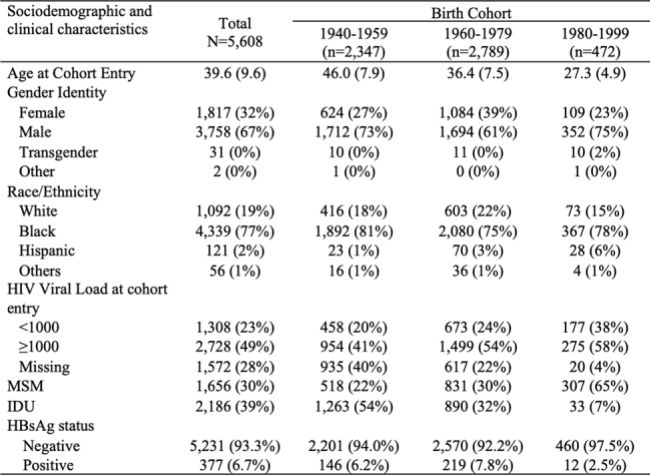
Baseline characteristics of patients by birth cohort

**Methods:**

We included PWH from the Johns Hopkins HIV Clinical Cohort who had HBsAg testing within one year of cohort entry. HBV infection was defined as a positive HBsAg test. Patients were categorized into birth cohorts of 1940-1959, 1960-1979 and 1980-1999 and then further dichotomized to pre- and post-1980 cohorts. Descriptive statistics were used to assess HBsAg prevalence by birth cohort and HIV infection risk factors [men who have sex with men (MSM) and injection drug use (IDU)]. Log binomial regression was used to assess the association of birth cohort (pre-1980 vs post-1980) with HBsAg positivity adjusting for race/ethnicity, HIV infection risk factors (MSM, IDU), baseline viral load, and year of and age at cohort entry.

**Figure 1.**
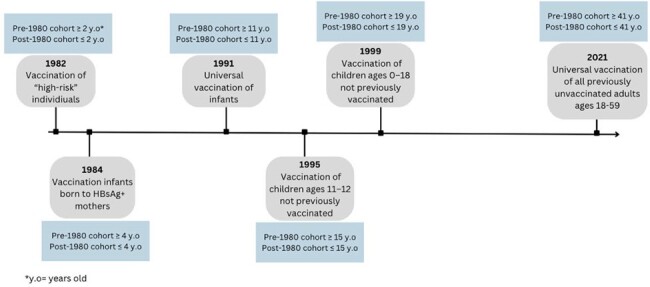
Timeline of HBV vaccination recommendations by the Advisory Committee of Immunization Practices and the corresponding age of patients in pre- and post-1980 birth cohort

**Results:**

Of the 5608 PWH enrolled in the cohort between 1993 and 2023, 77% were Black, 30% were MSM, and 39% had a history of IDU. Mean age at cohort entry was 39.6 (9.6). Overall HBV prevalence was 6.7% and prevalence by birth cohort was 6.2% (1940-1959), 7.8% (1960-1979) and 2.5% (1980-1999). Among MSM, HBV prevalence was higher at 10.6% (1940-1959), 11.4% (1960-1979) and 2.9% (1980-1999) compared to non-MSM at 5.0% (1940-1959), 6.3% (1960-1979) and 1.8% (1980-1999). HBV prevalence was similar in IDU and non-IDU groups. The risk of HBV infection was significantly lower in the post-1980 birth cohort vs the pre-1980 birth cohort (adjusted RR= 0.17; 95% CI 0.08, 0.34; adjusted RD= -0.06; 95% CI -0.07, -0.04).

**Conclusion:**

PWH born after 1980 had a lower risk of HBV co-infection than PWH born before 1980, highlighting the potential impact of HBV vaccination on HBV prevalence in PWH. PWH identifying as MSM continue to have disproportionately high HBV prevalence. Additional efforts are needed to implement universal HBV vaccination of adults as recommended in 2021.

**Disclosures:**

**Mark S. Sulkowski, MD**, AbbVie: Advisor/Consultant|Aligos Therapeutics: Advisor/Consultant|Gilead: Advisor/Consultant|GSK: Advisor/Consultant|GSK: Grant/Research Support|Janssen: Grant/Research Support|Precision Biosciences: Advisor/Consultant|Vir: Grant/Research Support|Virion: Advisor/Consultant **Oluwaseun Falade-Nwulia, MBBS ,MPH**, Abbvie Inc: Grant/Research Support|Gilead Sciences: Advisor/Consultant

